# Protective Effect of Anthocyanin-Enriched Polyphenols from *Hibiscus syriacus* L. (Malvaceae) against Ultraviolet B-Induced Damage

**DOI:** 10.3390/antiox10040584

**Published:** 2021-04-09

**Authors:** Wisurumuni Arachchilage Hasitha Maduranga Karunarathne, Ilandarage Menu Neelaka Molagoda, Kyoung Tae Lee, Yung Hyun Choi, Sang-Mi Yu, Chang-Hee Kang, Gi-Young Kim

**Affiliations:** 1 Department of Marine Life Science, Jeju National University, Jeju 63243, Korea; hasikarunarathne@gmail.com (W.A.H.M.K.); neelakagm2012@gmail.com (I.M.N.M.); 2 Department of Biosystems Technology, Faculty of Technology, University of Ruhuna, Matara 81000, Sri Lanka; 3 Forest Biomaterials Research Center, National Institute of Forest Science, Jinju 52817, Korea; leekt99@korea.kr; 4 Department of Biochemistry, College of Oriental Medicine, Dong-Eui University, Busan 47227, Korea; choiyh@deu.ac.kr; 5 Microbial Research Department, Nakdonggang National Institute of Biological Resources, Sangju 37242, Korea; smyu@nnibr.re.kr; 6 Bioresources Industrialization Support Department, Nakdonggang National Institute of Biological Resources, Sangju 37242, Korea; ckdgml3735@nnibr.re.kr

**Keywords:** *Hibiscus syriacus* L. (Malvaceae), ultraviolet B, reactive oxygen species, endoplasmic reticulum

## Abstract

Anthocyanin-enriched polyphenols from the flower petals of *H. syriacus* L. (Malvaceae, AHs) possess anti-septic shock, anti-oxidant, and anti-melanogenic properties. However, whether AHs positively or negatively regulate ultraviolet B (UVB)-mediated photoaging and photodamage remains unclear. This study aims to investigate the protective effect of AHs against UVB-induced damage. We examined the photoprotective effects of AHs on UVB-induced apoptosis, endoplasmic reticulum (ER) stress, and mitochondrial reactive oxygen species (mtROS). AHs prevented UVB irradiation-induced apoptosis of HaCaT keratinocytes by inhibiting caspase activation and ROS production. Moreover, AHs restored the survival rate and the hatchability of UVB-irradiated zebrafish larvae without any abnormalities. Furthermore, AHs inhibited UVB-induced ER stress, resulting in a decrease in mtROS production via the stabilization of the mitochondrial membrane potential. Our results indicate that AHs inhibit UVB-induced apoptosis by downregulating total cytosolic ROof cytosolic CaS and ER-mediated mitoROS production in both HaCaT keratinocytes and zebrafish larvae. These findings provide evidence for the applications of AHs to protect skin from UVB-induced photodamage.

## 1. Introduction

Excessive exposure to UV radiation is one of the most dangerous environmental factors in everyday people’s lives, causing photoaging, photodamage, carcinogenesis, and skin cell death [1]. UV radiation is divided into three subtypes based on wavelength: UVA (315–400 nm), UVB (280–315 nm), and UVC (100–280 nm). Of these three subtypes, 95% and 5% of UVA and UVB radiation reach the Earth’s surface, respectively, whereas UVC radiation cannot [2]. Nevertheless, UVA radiation can penetrate deeper into the dermis than UVB radiation, but UVB is more energetic than UVA, which enables both radiation subtypes to adversely impact on the epidermis. [3,4,5]. In particular, UVB radiation induces the overproduction of reactive oxygen species (ROS), which causes keratinocyte apoptosis through the stimulation of the caspase-mediated signaling pathway [6,7]. In this regard, antioxidants have been known to prevent keratinocytes from UV irradiation-induced photodamage [8].

The endoplasmic reticulum (ER) is a major organelle in eukaryotic cells and is involved in protein synthesis, folding, and posttranslational modifications, in addition to the quality control of secretory and transmembrane proteins [9,10]. Intrinsic or extrinsic disturbances cause the accumulation of misfolded proteins within the ER lumen and, in turn, the activation of an unfolded protein response (UPR) concomitant with accompanying ROS production, which induces the attenuation of protein synthesis and consequently triggers cell death [11,12]. A previous study showed that UVB-induced ROS production resulted in the ER stress response, along with transmission of Ca^2+^ from the ER lumen to mitochondria, causing a loss of the mitochondrial membrane potential and apoptosis [13]. This indicates that antioxidants can prevent cell damage and cell death from UVB radiation by inhibiting the ER stress response.

Absolute from *Hibiscus syriacus* L. (Malvaceae) flowers promoted the proliferation and wound healing of keratinocytes [14]. We previously reported that anthocyanin-enriched polyphenols from the petals of the *H. syriacus* L. (Malvaceae, AHs) potently inhibited melanogenesis in B16F10 melanoma cells and zebrafish larvae [15], and attenuated lipopolysaccharide-induced inflammation and endotoxic shock by inhibiting Toll-like receptor 4 (TLR4)/myeloid differentiation factor 2 (MD2)-mediated nuclear factor-κB (NF-κB) signaling pathway [16]. Recently, Molagoda and colleagues [17] also showed that AHs prevented cell death from oxidative stress by activating the nuclear factor erythroid 2-related factor 2 (Nrf2)/heme oxygenase-1 (HO-1) signaling pathway. However, whether AHs protect UVB-induced cell damage and death remains unclear. Therefore, in this study, we investigated the effect of AHs on apoptosis in HaCaT keratinocytes and the mortality and hatchability of zebrafish larvae against UVB irradiation by inhibiting ER stress-mediated ROS production.

## 2. Materials and Methods

### 2.1. Preparation of AHs

*H. syriacus* L. (Malvaceae) was cultivated in the *Hibiscus* clonal archive of the Korea Forest Research Institute, Suwon, Korea (N371505.56”, E12657016.11”) and identified by H.-Y. Kwon (National Institute of Forest Science, Suwon, Korea). Voucher specimens were deposited in the Korea Forest Service (NF-H8-F). AHs with a purity *(w/w)* over 95% were obtained in our previous study [15], which contained 17 anthocyanin-enriched polyphenols.

### 2.2. Reagents and Antibodies

Dulbecco’s Modified Eagle medium (DMEM), fetal bovine serum (FBS), antibiotic mixture, and trypsin-ethylenediaminetetraacetic acid (trypsin-EDTA) solution were obtained from WELGENE Inc. (Daegu, Korea). 3-(4,5-Dimethylthiazol-2-yl)-2,5-diphenyltetrazolium bromide (MTT), *N*-acetylcysteine (NAC), and salubrinal were purchased from Sigma Aldrich (St. Louis, MO, USA). 2ʹ,7ʹ-Dichlorodihydrofluorescein diacetate (DCFDA) was purchased from Molecular Probes (Eugene, OR, USA). Antibodies against caspase-3 (sc-65497), poly(adenosine diphosphate ribose) polymerase (PARP) (sc-365315), β-actin (sc-69879), CHOP (sc-575), and peroxidase-labeled anti-mouse immunoglobulins (sc-16102) were observed from Santa Cruz Biotechnology (Santa Cruz, CA, USA). Antibodies against GRP78 (PA1-014A), ATF4 (PA5-19521), eIF2α (PA5-27366), and p-eIF2α (MA5-15133) were purchased from Thermo Fisher Scientific (Waltham, MA, USA). MitoTracker Green, MitoSOX Red, and MitoTEMPO were also obtained from Thermo Fisher Scientific. Peroxidase-labeled anti-rabbit immunoglobulins (KO211708) was obtained from Koma Biotechnology (Seoul, Korea). All other chemicals were purchased from Sigma-Aldrich.

### 2.3. Cell Culture and Cell Viability

Human HaCaT keratinocytes were provided from American Type Cell Culture Collection (Manassas, VA, USA) and maintained in DMEM containing 10% FBS and antibiotic mixture. HaCaT keratinocytes (1 × 10^4^ cells/mL) were treated with various concentrations of AHs (0–800 µg/mL) for 24 h, and the MTT assay was performed. In a parallel experiment, cell images were captured by phase contrast microscopy (Macrotech, Goyang-si, Gyeonggi-do, Korea).

### 2.4. Analysis of Cell Viability and Population of Dead Cells

To analyze cell viability, dead cells, and total cell count, HaCaT keratinocytes were plated at a density of 1 × 10^4^ cells/mL and then treated with indicated concentrations of AHs (0–800 µg/mL) for 24 h. The cells were stained by a Muse Cell Count & Viability Kit (Luminex Corp., Austin, TX, USA) and analyzed by Muse Cell Analyzer (Luminex Corp.).

### 2.5. UVB Irradiation

Two different experimental protocols were performed: (1) HaCaT keratinocytes (80% confluent) were treated with AHs (0–400 µg/mL) for 2 h, then exposed by 30 mJ/cm^2^ UVB radiation (Bio-Link BLX-E312, Vilber Lourmat, Marne La Vallée, France), and cultured for 18 h (pretreatment), and (2) the cells were subjected to 30 mJ/cm^2^ UVB irradiation and then treated with (0–400 µg/mL) AHs for 18 h (posttreatment). All the radiation exposers were conducted at room temperature until the cells were exposed to 30 mJ/cm^2^.

### 2.6. Annexin V Staining for Apoptosis

HaCaT keratinocytes were seeded at a density of 1 × 10^4^ cell/mL. After pretreatment and post-treatment with AHs (0–400 µg/mL), the cells were incubated with a Muse Annexin V & Dead Cell Kit (Luminex Corp.), and then apoptotic cell populations were measured by a Muse Cell Analyzer.

### 2.7. Analysis of Caspase-3/7 Activity

HaCaT keratinocytes were treated with various concentrations of AHs (0–400 µg/mL) for 2 h. UVB (30 mJ/cm^2^) was irradiated and then cultured for 18 h. Then, the cells were washed with ice-cold PBS and stained by a Muse Caspase-3/7 Kit (Luminex Corp.). Caspase-3/7^+^ cell populations were measured using the Muse Cell Analyzer.

### 2.8. Analysis of Intracellular Reactive Oxygen Species (ROS)

HaCaT keratinocytes were treated with AHs (0–400 µg/mL) for 2 h and then exposed to UVB (30 mJ/cm^2^). Three hours after UVB irradiation, the cells were incubated with a Muse Oxidative Stress Kit (Luminex Corp.) for 30 min. ROS^+^ cell populations were measured by the Muse Cell Analyzer.

### 2.9. Mitochondrial Membranes Potential and Mitochondrial ROS (mtROS)

HaCaT keratinocytes were seeded at a density of 1 × 10^4^ cells/mL and then treated with AHs (0–400 µg/mL) for 2 h prior to UVB irradiation (30 mJ/cm^2^). Three hours after UVB irradiation, the cells were incubated with a Muse Mitopotential Kit (Luminex Corp.), and then mitochondrial membrane potential was measured by the Muse Cell Analyzer. In a parallel experiment, the cells were stained with 0.5 µM MitoTracker Green for 30 min and then with 2 µM MitoSOX Red for 10 min in the presence and absence of MitoTEMPO. The image was captured by a CELENA S Digital Imaging System (LogosBiosystem, Anyang, Gyeonggido, Korea).

### 2.10. Western Blotting

HaCaT keratinocytes were pretreated with AHs (0–400 μg/mL) for 2 h prior to UVB irradiation (30 mJ/cm^2^), and kept in AHs–free cell culture medium for 18 h. Total cellular protein was prepared using RIPA lysis buffer (iNtRON Biotechnology, Seongnam, Gyeonggido, Korea). Proteins of interest were immunoblotted using the indicated primary (1:1000 dilution) and secondary antibodies (1:10,000 dilution). The images were captured by ImageQuant LAS 500 (GE Healthcare Bio-Sciences AB, Uppsala, Sweden). The expressional values of each protein were normalized to the level of β-actin.

### 2.11. Maintenance of Zebrafish

AB strain zebrafish were served from C.H. Kang (Nakdong National Institute of Biological Resources, Sangju, Gyeongsangbukdo, Korea) and handled according to standard guidelines of the Animal Care and Use Committee of Jeju National University (Jeju-si, Jeju Special Self-governing Province, Korea; approval no.: 2020-0018). Embryos were cultured in embryo medium supplemented with 1% methylene blue at 28 °C.

### 2.12. Evaluation of Survival Rate and Hatchability in UVB-Irradiated Zebrafish Larvae and Embryos

For the analysis of survival rate, two days postfertilization (dpf) zebrafish larvae (*n* = 20) were treated with AHs (0–200 μg/mL) for 2 h and then irradiated by UVB (150 mJ/cm^2^). Survival rate and morphological abnormality were monitored for 24 h. For analysis of hatchability, 1 dpf unhatched zebrafish embryos (*n* = 20) were treated with AHs (0–200 μg/mL) for 2 h and irradiated by UVB (150 mJ/cm^2^). Hatchability of zebrafish embryos was monitored by 5 dpf. Distance to the zebrafish from the UVB source was 30 cm while all the radiation exposers were conducted at room temperature until the cells were exposed to 30 mJ/cm^2^.

### 2.13. Detection of Total ROS and mitoROS in Zebrafish Larvae

Intracellular ROS production was determined in zebrafish larvae after UVB irradiation using a fluorescent probe, DCFDA. Briefly, 2 dpf zebrafish larvae (*n* = 20) were treated with AHs (0–200 μg/mL) for 2 h and then irradiated by UVB (150 mJ/cm^2^). To analyze total intracellular ROS production, the larvae were stained with 20 µM DCFDA 1 h after UVB irradiation. For analysis of mtROS production, the larvae were stained with 10 µM MitoSOX Red for 30 min and 5 µM MitoTracker Green for 10 min in embryo medium on the presence and absence of 10 μM MitoTEMPO and then washed twice with fresh media. The larvae were anesthetized in 0.04% tricaine methanesulfonate solution and fluorescence images were captured by a CELENA S Digital Imaging System.

### 2.14. Statistical Analysis

The images of western blotting were visualized by ImageQuant LAS 500 and transported into Adobe Photoshop. All data represented as the mean ± the standard error of the median (SEM). Statistical analysis was performed on the Sigma Plot 12.0 by the Student’s *t*-test and unpaired one-way analysis of variance (ANOVA) with the Bonferroni correction. Statistical significance was set at * *p* < 0.05, ** *p* < 0.01, *** *p* < 0.001, and ^###^ *p* < 0.001.

## 3. Results

### 3.1. Low Concentrations of AHs Are Not Cytotoxic in HaCaT Keratinocytes

**Figure 1:**
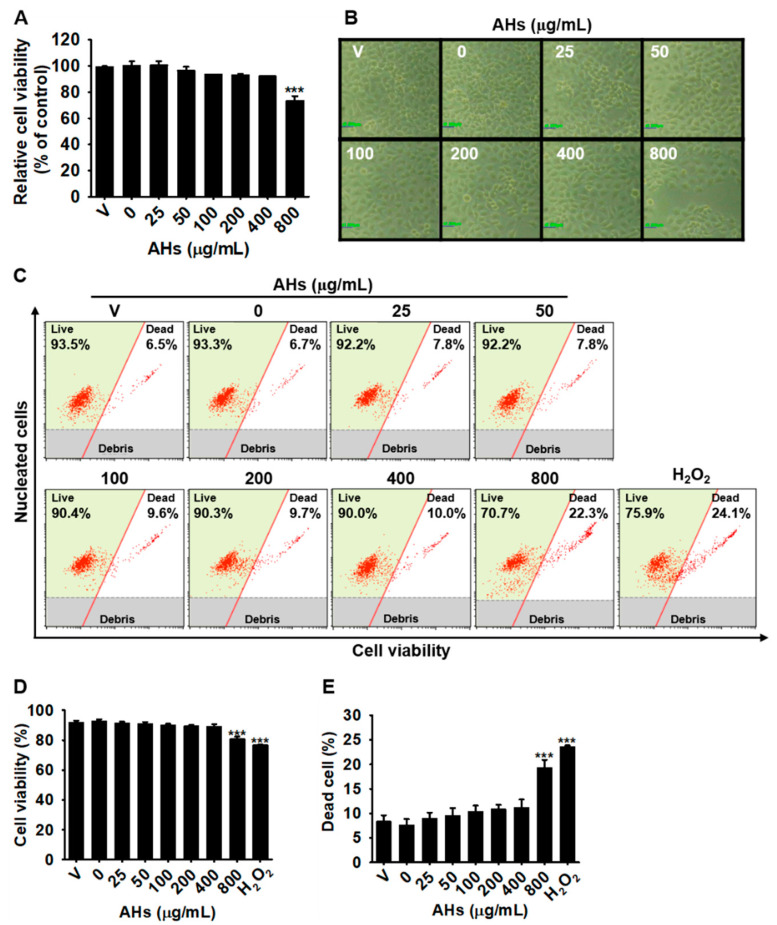
Cytotoxic effect of anthocyanins from the flower petals of *Hibiscus syriacus* L. (Malvaceae, AHs). (**A**) Relative cell viability and (**B**) cellular morphology (×20). Scare bare = 20 μm. (**C**) Flow cytometry analysis was performed. (**D**) Cell viability (%) and (**E**) dead cell population (%) were represented from the flow cytometry data. *** *p* < 0.001 vs. untreated cells. V, vehicle control (0.01% DMSO).

To evaluate the effect of AHs on the viability of HaCaT keratinocytes, we treated the cells with the indicated concentrations of AHs (0–800 μg/mL) for 24 h, and the MTT assay was performed. The concentration of 800 μg/mL AHs significantly decreased the cell viability to 73.4 ± 3.4% compared with that observed in the untreated cells, and no cytotoxicity was observed below 400 μg/mL AHs (Figure 1A). Consistent with the MTT data, only the highest concentration (800 μg/mL) of AHs reduced cell number compared with that of the untreated cells, and no morphological change was seen with the other concentrations of AHs (Figure 1B). To further confirm the effect of AHs on cell viability, the total cell count, and the dead cell population, flow cytometry was performed under the same experimental conditions (Figure 1C). The concentration of 800 μg/mL AHs significantly decreased cell viability to 80.7 ± 1.6% (Figure 1D) and increased the dead cell population to 19.3 ± 1.6% (Figure 1E) compared with those of the untreated cells (96.5 ± 1.3% cell viability, (18.8 ± 1.3) × 10^5^ cells/mL total cell count, and 7.2 ± 1.3% dead cell population, respectively), which were the same as those in H_2_O_2_-treated cells. However, below 400 μg/mL of AHs, cell viability and the total cell count remained similar to those of the untreated cells, with no increase in the dead cell population. Therefore, low concentrations (≤400 μg) of AHs were selected for further study.

### 3.2. Pretreatment, but Not Posttreatment with AHs Protects HaCaT Keratinocytes from UVB-Induced Apoptosis

**Figure 2:**
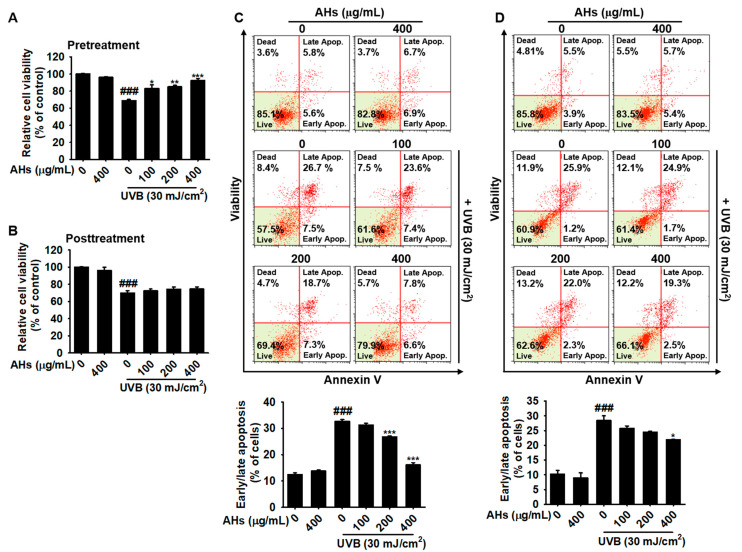
Antiapoptotic effect of anthocyanins from the flower petals of *Hibiscus syriacus* L. (Malvaceae, AHs) in UVB-irradiated HaCaT keratinocytes. (**A**,**B**) Relative cell viability. (**C**,**D**) Distribution (top) and population (bottom) of early/late apoptotic cells. * *p* < 0.05, ** *p* < 0.01, and *** *p* < 0.001 vs. UVB-irradiated cells and *^###^ p* < 0.001 vs. untreated cells.

Next, we investigated whether AHs protected HaCaT keratinocytes from UVB-induced apoptosis. Data from the MTT assay showed that UVB irradiation significantly decreased the viability to 68.8 ± 1.4% compared with that observed in the untreated cells. However, when the cells were pretreated with AHs, relative viability was restored in a concentration-dependent manner (81.0 ± 4.3%, 85.0 ± 1.8%, and 92.2 ± 2.2% at 100, 200, and 400 μg/mL of AHs, respectively, Figure 2A), indicating that pretreatment with AHs has cytoprotective effects against UVB-induced cytotoxicity. However, posttreatment with AHs did not recover UVB-induced cytotoxicity (Figure 2B). To identify whether AHs prevented UVB-induced apoptosis, HaCaT keratinocytes were stained using a Muse Annexin & Dead Cell Kit (Figure 2C,D, top). Flow cytometry data revealed that UVB irradiation induced an increase in apoptosis (32.6 ± 0.8%); however, pretreatment with AHs significantly downregulated the proportion of UVB-induced apoptotic cells at 200 and 400 μg (25.8 ± 0.3% and 16.1 ± 0.9%, respectively; Figure 2C, bottom). However, posttreatment with the highest concentration (400 μg/mL) of AHs slightly decreased UVB-induced apoptosis from 28.5 ± 1.6% to 21.9 ± 0.2% (Figure 2D, bottom). These data indicate that pretreatment with AHs is a potential barrier to UVB-induced apoptosis in HaCaT keratinocytes, but posttreatment with AHs is not effective.

### 3.3. AHs Prevents UVB-Induced Apoptosis by Inhibiting the Caspase-Mediated Signaling Pathway

**Figure 3:**
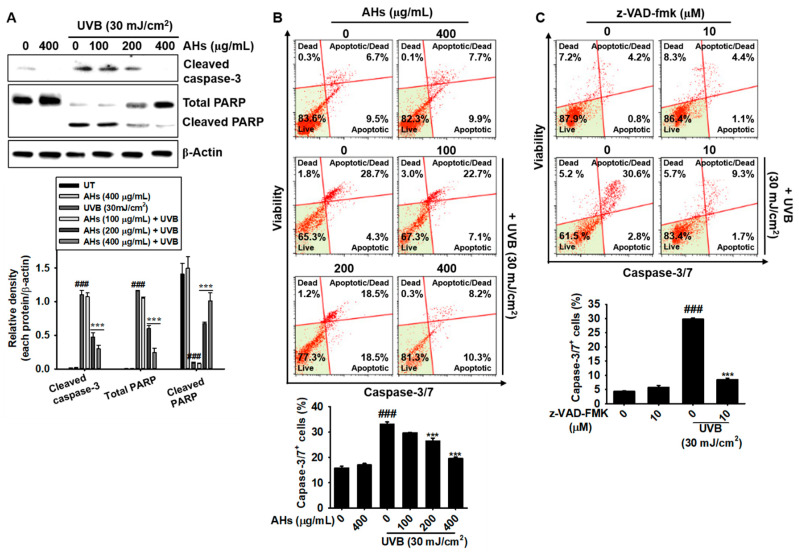
Inhibition of the caspase signaling pathway by anthocyanins from the flower petals of *Hibiscus syriacus* L. (Malvaceae, AHs) in UVB-irradiated HaCaT keratinocytes. (**A**) Caspase-3 and PARP protein expression (top) and relative density (bottom). (**B**,**C**) Distribution (top) and population (bottom) of caspase-3/7^+^ apoptotic cells. *** *p* < 0.001 vs. UVB-irradiated cells and ^###^ *p* < 0.001 vs. untreated cells.

Next, we investigated whether AHs prevented UVB-mediated apoptosis by inhibiting apoptosis-regulating key molecules such as PARP and caspases. As shown in Figure 3A, UVB irradiation significantly increased the expression of cleaved caspase-3 and the consequent cleavage of PARP; however, all concentrations of AHs in this experiment inhibited the UVB-induced cleavage of capase-3 and PARP, except for the lowest concentration of AHs (100 μg/mL). Furthermore, we confirmed whether the antiapoptotic effect of AHs is regulated through the caspase-3/7 signaling pathway under exposure to UVB radiation. Flow cytometry data showed that UVB irradiation increased the total population of caspase-3/7^+^ apoptotic HaCaT keratinocytes from 16.2 ± 0.4% to 33.1 ± 0.9%, which was significantly reduced in the presence of AHs (26.4 ± 1.2% and 19.6 ± 0.6% at 200 and 400 μg/mL AHs, respectively); however, the anti-apoptotic effect of AHs was not observed at 100 μg/mL (Figure 3B). To investigate whether AHs inhibited UVB-induced apoptosis by inhibiting the caspase signaling pathway, a pan-caspase inhibitor, z-VAD-FMK, was pretreated before UVB irradiation, and caspase-3/7^+^ apoptotic cell populations were measured using flow cytometry. UVB irradiation increased caspase-3/7^+^ apoptotic cells to 29.8 ± 0.4%; however, pretreatment with z-VAD-FMK significantly downregulated the proportion of caspase-3/7^+^ apoptotic cells (8.5 ± 0.4%, Figure 3C). Collectively, these data indicate that AHs suppress caspase-3/7 activity, resulting in the reduction of UVB-induced apoptosis in HaCaT keratinocytes.

### 3.4. AHs Decreases Mortality and Morphological Abnormalities in UVB-Irradiated Zebrafish Larvae and Increases Hatchability in UVB-Irradiated Zebrafish Embryos

**Figure 4:**
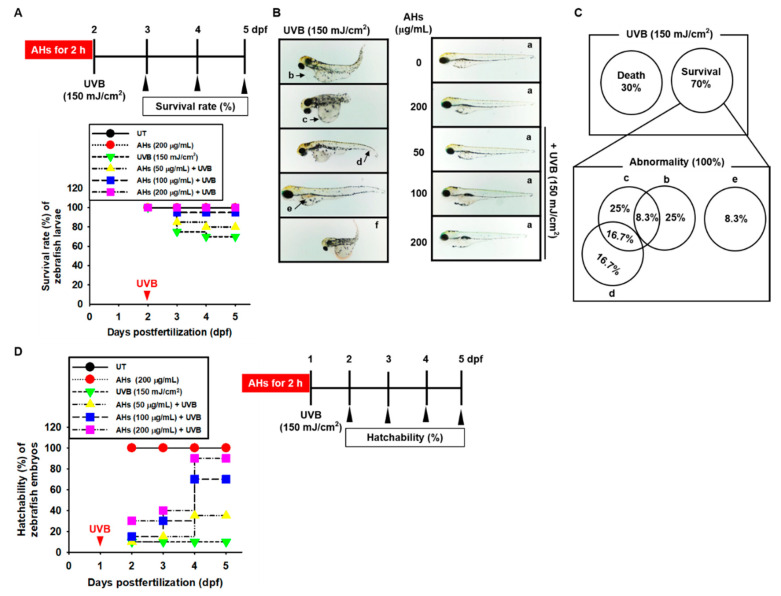
Effect of anthocyanins from the flower petals of *Hibiscus syriacus* L. (Malvaceae, AHs) on mortality and morphological abnormalities in UVB-irradiated zebrafish larvae, and hatchability in UVB-irradiated zebrafish embryos. (**A**) Survival rate and (**B**) morphological abnormalities in UVB irradiation (left) and AHs plus UVB (right). (**C**) The morphological abnormality percentages of surviving larvae. (**D**) Hatchability rate (%). UT, untreatment.

Zebrafish has been considered as an ideal model organism to screen the UVB-protective drugs due to their ability to absorb UV spectrum, optical clearance nature, and rapid development and growth rate. To assess the effect of AHs on the UVB-induced mortality and morphological abnormalities of zebrafish larvae, 2 dpf larvae were treated with AHs (0–200 μg/mL) for 2 h and then exposed to 150 mJ/cm^2^ UVB radiation (Figure 4A, top). At 5 dpf after UVB irradiation, approximately 30% of the zebrafish larvae had died (Figure 4A), and all remaining larvae (70%) showed severe morphological abnormalities (Figure 4B, left). Of these, 25% corresponded to pericardial edema (b), 25% to yolk sac edema (c), and 8.3% to hemorrhagic lesions (e; Figure 4C). In addition, some UVB-irradiated zebrafish larvae showed more than one morphological abnormality such as 8.3% pericardial edema (b) plus yolk sac edema (c), and 16.7% yolk sac edema (c) plus shortened tails (g; Figure 4B, left, and Figure 4C). In contrast, treatment with 200 μg/mL AHs completely blocked UVB-induced mortality and morphological abnormalities in the larvae (Figure 4A,B, right). A concentration of 100 μg/mL AHs inhibited UVB-induced mortality (1 out of 20 larvae died) by increasing the survival rate to approximately 95%; however, the lowest concentration of AHs at 50 μg/mL increased the survival rate from 70% to 80%, and the remaining zebrafish larvae did not show any abnormalities, which indicates that AHs diminished severe mortality and morphological abnormalities in UVB-irradiated zebrafish larvae. Furthermore, we assessed whether AHs could be used to overcome the UVB-induced inability to hatch in zebrafish embryos. One dpf embryos were treated with AHs (0–200 μg/mL) for 2 h and then exposed to 150 mJ/cm^2^ UVB radiation. As shown in Figure 4D, only 10% of zebrafish embryos hatched in UVB-irradiated conditions at 5 dpf. However, treatment with AHs at 200 μg/mL gradually increased hatchability and reached 90% at 4 dpf. Low concentrations of AHs also increased hatchability at 4 dpf (35% and 70% at 50 μg/mL and 100 μg/mL, respectively). These data indicate that AHs attenuate severe mortality and morphological abnormalities in UVB-irradiated zebrafish larvae, and restores hatchability in UVB-irradiated zebrafish embryos.

### 3.5. AHs Prevents UVB-Induced Apoptosis in HaCaT Keratinocytes and Mortality in Zebrafish Larvae by Inhibiting Intracellular ROS Production

**Figure 5:**
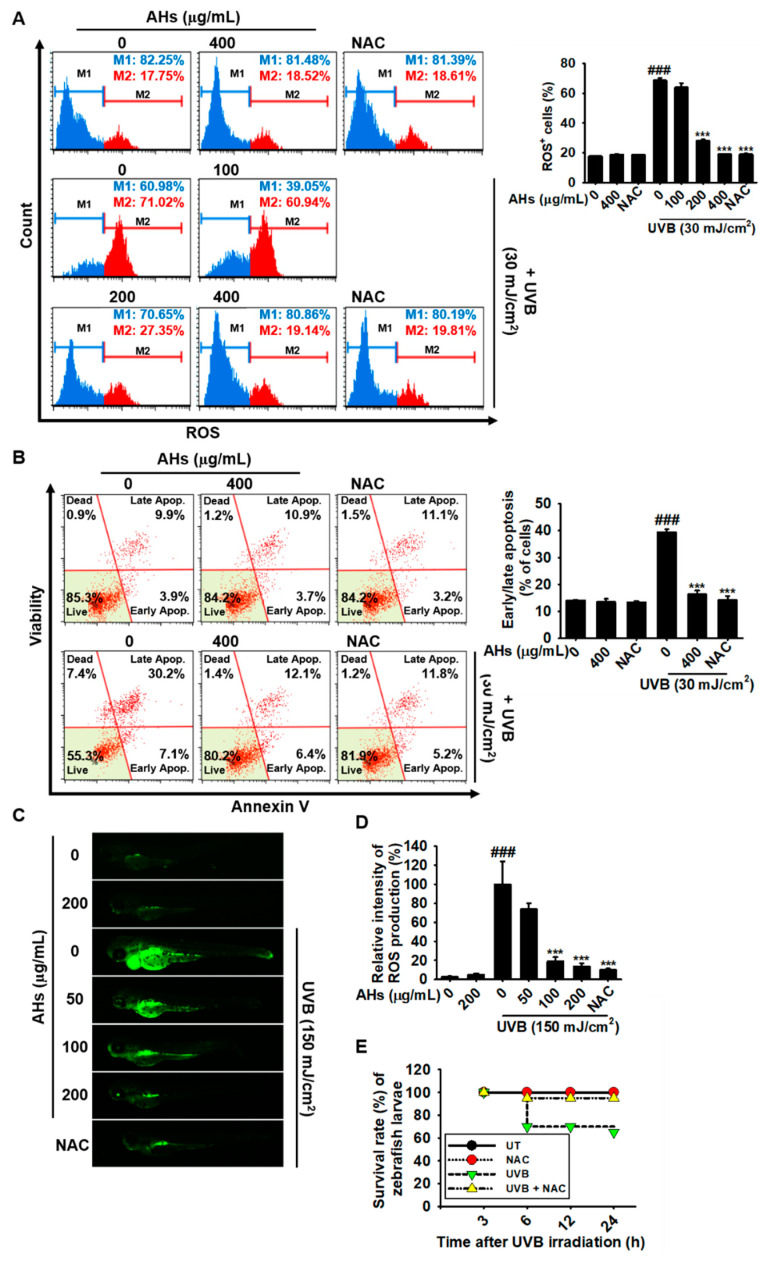
Effect of anthocyanins from the flower petals of *Hibiscus syriacus* L. (Malvaceae, AHs) on UVB-induced ROS production, cytotoxicity, and mortality. (**A**) Intracellular ROS production (left) and ROS^+^ cell populations (right). (**B**) Distribution (left) and population (*r*) of early/late apoptotic cells. (**C**) DCFDA fluorescence images and (**D**) intensity. (**E**) Survival rate of zebrafish larvae. *** *p* < 0.001 vs. UVB-irradiated cells or zebrafish larvae and ^###^ *p* < 0.001 vs. untreated cells or zebrafish larvae. UT, untreatment.

We monitored whether AHs downregulated UVB-induced intracellular ROS production. Flow cytometry data revealed that UVB irradiation significantly increased intracellular ROS^+^ HaCaT keratinocytes to 68.8 ± 1.5% (Figure 5A). However, treatment with AHs potently reduced the UVB-induced ROS^+^ cell population in a concentration-dependent manner (28.0 ± 0.9% and 19.1 ± 0.1% at 200 and 400 μg/mL AHs, respectively). The highest concentration of AHs (400 μg/mL) reduced UVB-induced ROS^+^ cell numbers to the same degree as those observed in the NAC-treated cells (19.0 ± 0.5%) and the untreated cells (19.0 ± 0.5%); however, the lowest concentration of AHs (100 μg/mL) did not show any significant downregulation of ROS^+^ cell numbers (64.0 ± 2.9%). In addition, NAC treatment markedly reduced UVB-induced apoptosis from 39.4 ± 0.5% to 14.3 ± 1.4%, which was almost the same as that observed in the AHs-treated cells (16.1 ± 1.5%, Figure 5B). To further confirm the in vivo effect of AHs against UVB-induced oxidative stress, we measured intracellular ROS production in zebrafish larvae (Figure 5C). ROS production in UVB-irradiated zebrafish larvae remarkably increased (Figure 5C) and reached 100.0 ± 23.9% compared with that in non-irradiated larvae (5.5 ± 1.3%, Figure 5D). Treatment with AHs downregulated the UVB-induced ROS fluorescence intensity in a concentration-dependent manner (74.0 ± 6.1%, 18.9 ± 4.6%, and 13.5 ± 3.2% at 50, 100, and 200 μg/mL AHs, respectively; Figure 5C,D). As expected, UVB irradiation significantly decreased the survival rate of zebrafish larvae to approximately 65% at 24 h; however, treatment with NAC nearly restored the survival rate (Figure 5E), indicating that excessive ROS production enhances the UVB-induced death of zebrafish larvae and that the antioxidant effect of AHs inhibits UVB-induced apoptosis and mortality. Altogether, these data indicate that AHs downregulate UVB-induced intracellular ROS generation in both HaCaT keratinocytes and zebrafish larvae, resulting in the inhibition of oxidative stress-mediated apoptosis and mortality.

### 3.6. AHs Decreases the Depolarization of the Mitochondrial Membrane Potential and mtROS Production in UVB-Irradiated HaCaT Keratinocytes

**Figure 6:**
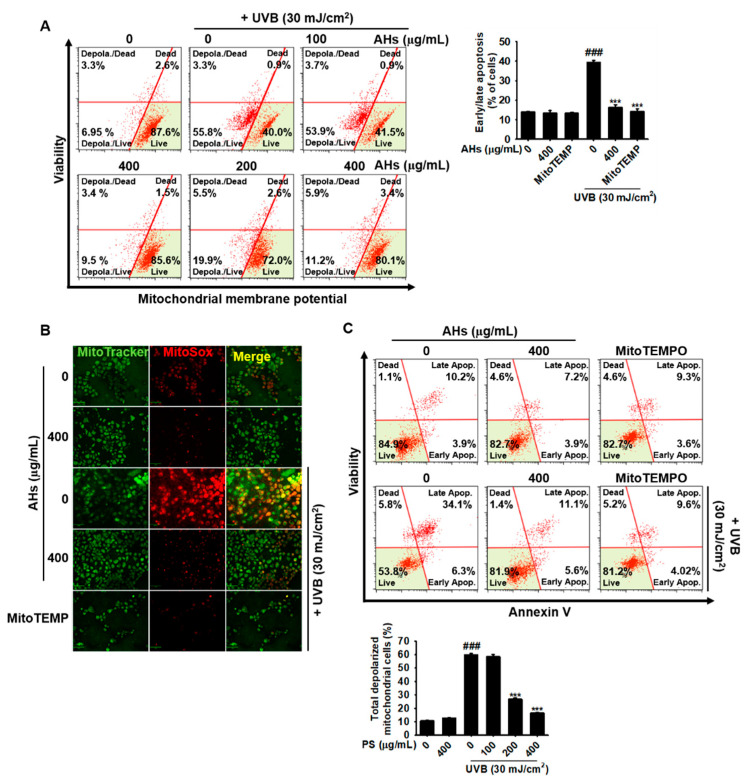
Effect of anthocyanins from the flower petals of *Hibiscus syriacus* L. (Malvaceae, AHs) on UVB-induced depolarization of mitochondrial membrane potential and subsequent mtROS production. (**A**) Depolarization (left) and mean percentage (right) of the mitochondrial membrane potential. (**B**) Fluorescence of MitoTracker Green and MitoSOX Red. (**C**) Distribution (top) and population (bottom) of early/late apoptotic cells. *** *p* < 0.001 vs. UVB-irradiated cells and ^###^ *p* < 0.001 vs. untreated cells.

Next, we investigated the potential effect of AHs on the depolarization of mitochondrial membrane potential and mtROS production in UVB-irradiated HaCaT keratinocytes. Flow cytometry data showed that UVB irradiation resulted in a significant increase in the total number of mitochondria with depolarized membrane potential in HaCaT keratinocytes from 9.4 ± 0.8% to 59.8 ± 1.0%, and AHs markedly reduced the number of mitochondria with depolarized membrane potential in a concentration-dependent manner (58.4 ± 1.6%, 26.7 ± 1.0%, and 16.7 ± 0.4% at 100, 200, and 400 μg/mL AHs, respectively; Figure 6A). Immunofluorescence staining also revealed that UVB irradiation significantly increased mtROS (MitoSOX Red) production in the mitochondria (MitoTracker Green); however, AHs completely suppressed UVB-induced mtROS production (Figure 6B), which was comparable to that observed in the MitoTEMPO-treated cells. Furthermore, MitoTEMPO significantly reduced UVB-induced apoptosis from 41.8 ± 0.7% to 13.8 ± 1.4%, which was similar to the levels of the untreated (14.3 ± 1.7%) and the 400 μg/mL AHs-treated cells (12.1 ± 1.3%, Figure 6C). These results indicate that AHs stabilizes the mitochondrial membrane potential against UVB irradiation and subsequently reduces excessive mtROS production, leading to the prevention of apoptosis.

### 3.7. AHs Reduces mtROS Production in UVB-Irradiated Zebrafish Larvae, Resulting in an Increase in the Survival Rate

**Figure 7:**
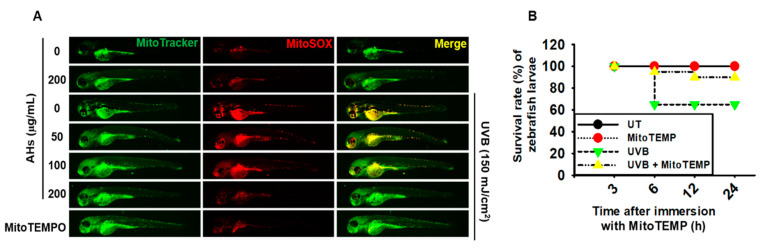
Effect of anthocyanins from the flower petals of *Hibiscus syriacus* L. (Malvaceae, AHs) on mtROS production and the survival rate of UVB-irradiated zebrafish larvae. (**A**) Staining of MitoSOX Red and MitoTracker Green. (**B**) Survival rate of zebrafish larvae. UT, untreatment.

To further confirm the effect of AHs on mtROS production and mortality in UVB-irradiated zebrafish larvae, we stained the larvae with MitoSOX Red (mtROS) and MitoTracker Green (mitochondria). As shown in Figure 7A, UVB irradiation markedly induced mtROS production in the larvae; however, treatment with AHs significantly reduced UVB-induced mtROS production in a concentration-dependent manner. MitoTEMPO also suppressed UVB-induced mtROS production in zebrafish larvae and the inhibitory levels were comparable to the effect of AHs at 200 μg/mL. In addition, UVB irradiation significantly decreased the survival rate of zebrafish larvae to approximately 65% and MitoTEMPO effectively prevented mortality and increased the survival rate to approximately 90% (Figure 7B). These data indicate that AHs reduces the excessive production of UVB-induced mtROS in zebrafish larvae, resulting in reduced mortality.

### 3.8. AHs Inhibits UVB-Induced ER Stress and Consequently Increases the Survival Rate of Zebrafish Larvae through the Inhibition of mtROS Production

**Figure 8:**
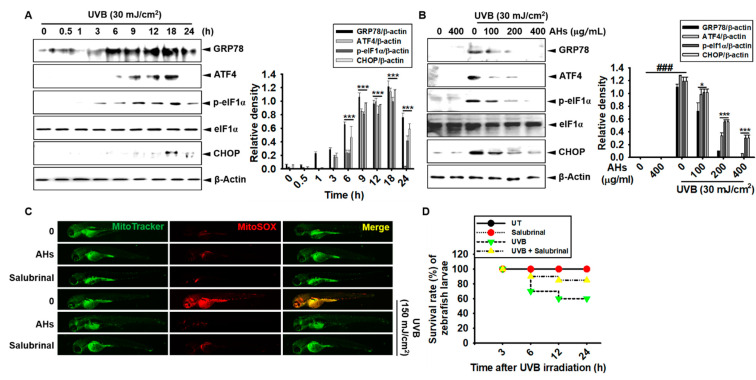
Effect of anthocyanins from the flower petals of *Hibiscus syriacus* L. (Malvaceae, AHs) on ER stress and mtROS production. (**A**) and (**B**) The expression of GRP78, ATF4, p-eIF1α, CHOP, and β-actin protein (left) and relative density (right). (**C**) The staining of MitoSOX Red and MitoTracker Green. (**D**) Survival rate of zebrafish. * p < 0.05 and *** *p* < 0.001 vs. UVB-irradiated cells and ^###^ *p* < 0.001 vs. untreated cells (UT).

To evaluate the effect of AHs on UVB-induced ER stress, western blotting was performed to check the expression of ER stress marker proteins. UVB-irradiated HaCaT keratinocytes significantly increased the expression of GRP78, ATF4, p-eIF1α, and CHOP; GRP78, ATF4, and p-eIF1α expression increased from 6 h to 18 h and then gradually decreased at 24 h (Figure 8A). CHOP was expressed at 18 h, and its expression partially decreased at 24 h. However, AHs decreased the expression of ER stress marker proteins in a concentration-dependent manner 18 h after UVB irradiation in HaCaT keratinocytes; in particular, 400 μg/mL AHs strongly inhibited the expression of GRP78, ATF4, p-eIF1α, and CHOP (Figure 8B). Next, to determine whether the inhibition of ER stress downregulates UVB-irradiated mtROS production, 2 dpf zebrafish larvae were treated with an ER stress inhibitor, salubrinal, 2 h before UVB irradiation, and the ER stress was compared with that in the AHs-treated larvae. As expected, mtROS production did not increase in the untreated larvae as well as in the AHs- and salubrinal-treated larvae; however, a dramatic increase in mtROS production was observed in UVB-irradiated larvae (Figure 8C). Salubrinal also reduced UVB-induced mtROS generation in the larvae, which was comparable to that observed in the AHs-treated larvae. In addition, UVB irradiation decreased the survival rate of zebrafish larvae to approximately 60%, and salubrinal strongly restored the survival rate to approximately 85% (Figure 8D), which was similar to that observed in AHs-treated larvae (Figure 4A). Collectively, our results indicate that UVB irradiation induces ER stress and as a consequent increase in mtROS production, causing an increase in the mortality of zebrafish larvae, which was markedly reduced in the presence of AHs.

## 4. Discussion

Recently, Yang and colleagues [18] revealed that both the water extract and enzyme lysate of the root and stem bark of *H. syriacus* L. (Malvaceae) protect the skin from chronic UVB-induced photoaging and photodamage via an enhancement of skin hydration and collagen synthesis. In particular, hexane extract of *H*. *syriacus* L. (Malvaceae) flowers showed more protective activity against UVB-induced inflammation than extracts of other plant parts such as cortexes, roots, leaves, and seeds [18]. Nevertheless, the precise mechanism underlying the photoprotective effects of *H*. *syriacus* L. (Malvaceae) flower extract against UVB irradiation has not been elucidated. Previously, we purified AHs from *H*. *syriacus* L. (Malvaceae) flower petals, which contained 17 anthocyanins (cyanidin-3-*O*-galactose, cyanidin-3-*O*-glucoside, orientin-7-*O*-glucoside, cyanidin-3,5-*O*-diglucoside, isoorientin-4ʹ-*O*-glucoside, isovitexin-4ʹ-*O*-glucoside, vitexin-4ʹ-*O*-glucoside-2″-*O*-rhamnoside, isovitexin-7-*O*-glucoside, apigenin-8-C-β-D-glucopyranoside, isovitexin-2″-*O*-rhamnoside, apigenin-6-C-β-D-glucopyranoside, apigenin-6-C-glucoside-7-(6″-*O*-acetyl)-glucoside, kaempferol-*O*-glucoside derivative, kaempferol-7-*O*-glucoside, kaempferol-3-*O*-glucoside, apigenin-7-*O*-glucoside, and kaempferol-3-(6″-acetylglucoside)) that possessed antimelanogenic [15], antiseptic shock [16], and antioxidant activity [17]. In addition, in this study, we found that AHs showed photoprotective activity against UVB irradiation by inhibiting ER stress-mediated mtROS production.

UVB radiation is mostly absorbed in the epidermis and can cause skin disorders such as skin cell death and skin malignancies by resulting in ROS-mediated oxidative damage to DNA [19]. Antioxidants significantly decrease UVB-induced keratinocyte apoptosis, whereas ROS inducers have been found to elevate the UVB-mediated apoptotic signaling pathway [20,21,22]. In this study, we found that pretreatment with AHs alleviates UVB-induced apoptosis in HaCaT keratinocytes by suppressing intracellular ROS production. Moreover, UVB irradiation increased the mortality of zebrafish larvae and decreases the hatchability of zebrafish embryos, whereas treatment with AHs gradually restored the survival rate and hatchability, along with downregulation of intracellular ROS production, indicating that AHs function as an antioxidant to prevent UVB-induced ROS production. In particular, UVB irradiation enhanced cytosolic ROS production [23,24,25] while we have previously confirmed that AHs could diminish the cytosolic ROS by activating the Nrf2-mediated HO-1 pathway under the H_2_O_2_-induced cytotoxic conditions [17]. Aligning with previous findings, AHs potently inhibited the UVB-induced photodamage through sequestering the cytosolic and mitochondrial ROS, however, the exact anthocyanins involved among the 17 anthocyanin found in AHs remain to be discovered. Therefore, AHs can be used to protect the skin from UVB-induced photodamage.

The ER is a large membrane-enclosed cellular organelle that is involved in the synthesis, folding, maturation, and transport of proteins [26]. Mera et al. [27] revealed that UVB irradiation promotes ER stress-mediated apoptosis in keratinocytes. In addition, Ji and colleagues [28] found that salubrinal, an ER stress inhibitor, protects skin fibroblasts from UVB-induced apoptosis by blocking ER stress and the level of cytosolic Ca^2+^ [28]. In this study, we found that both AHs and salubrinal inhibit the expression of ER stress marker proteins in UVB-irradiated HaCaT keratinocytes and significantly attenuates the UVB-induced mortality of zebrafish larvae. In addition, during ER stress, the release of Ca^2+^ from the ER lumen subsequently triggers the rapid uptake of Ca^2+^ in mitochondria through the mitochondrial Ca^2+^ uniporter, causing mtROS-mediated cell death concomitant with depolarization of the mitochondrial membrane potential [29]. We also found that AHs stabilizes the UVB-induced depolarization of the mitochondrial membrane potential and subsequently reduces mtROS production, resulting in cytoprotective activity and an increase in the survival rate of zebrafish larvae. These results suggest that AHs blocks apoptosis in UVB-irradiated keratinocytes and increases the survival rate of UVB-irradiated zebrafish larvae and the hatchability of UVB-irradiated zebrafish embryos by inhibiting ER stress-mediated mtROS production. Nevertheless, whether AHs is effective against UVB-induced photodamage in humans will need to be evaluated. In addition, the specific anthocyanins involved in AHs-mediated cytoprotective effects need to be identified since it will eventually help to replace the current use of UV-filters such as 2-4,dihydroxybenzophenone-1 in the cosmetics industry.

## 5. Conclusions

In conclusion, our results show that AHs protects HaCaT keratinocytes and zebrafish from UVB irradiation-induced damage by inhibiting ER stress-mediated mtROS production. Therefore, AHs could be a novel drug candidate for protection against UVB-induced photoaging and photodamage.

## Data Availability

The data presented in this study are available on request from the corresponding author.
